# Supplementary material for: Revealing hidden spin polarization in centrosymmetric van der Waals materials on ultrafast timescales
Source: Nat Commun. 2024 Apr 27;15:3573. doi: 10.1038/s41467-024-47821-4 (PMC11055871; doi:10.1038/s41467-024-47821-4)
Supplement: Supplementary file 1 — Supplementary information [file 41467_2024_47821_MOESM1_ESM.pdf]

Revealing Hidden Spin Polarization in Centrosymmetric van der Waals Materials on Ultrafast Timescales

Supplementary Information

B. Arnoldi[1], S. L. Zachritz[2], S. Hedwig[1], M. Aeschlimann[1], O.L.A. Monti[2,3*], B. Stadtmüller[1,4#]

1 Department of Physics and Research Center OPTIMAS, Rheinland-Pfälzische Technische Universität Kaiserslautern-Landau, Erwin-Schroedinger-Strasse 46, Kaiserslautern 67663, Germany

2 Department of Chemistry and Biochemistry, University of Arizona, Tucson, Arizona 85721, United States

3 Department of Physics, University of Arizona, Tucson, Arizona 85721, United States

4 Institute of Physics, Johannes Gutenberg University Mainz, Staudingerweg 7, 55128 Mainz, Germany

* monti@arizona.edu

# b.stadtmueller@rptu.de

Supplementary Figures

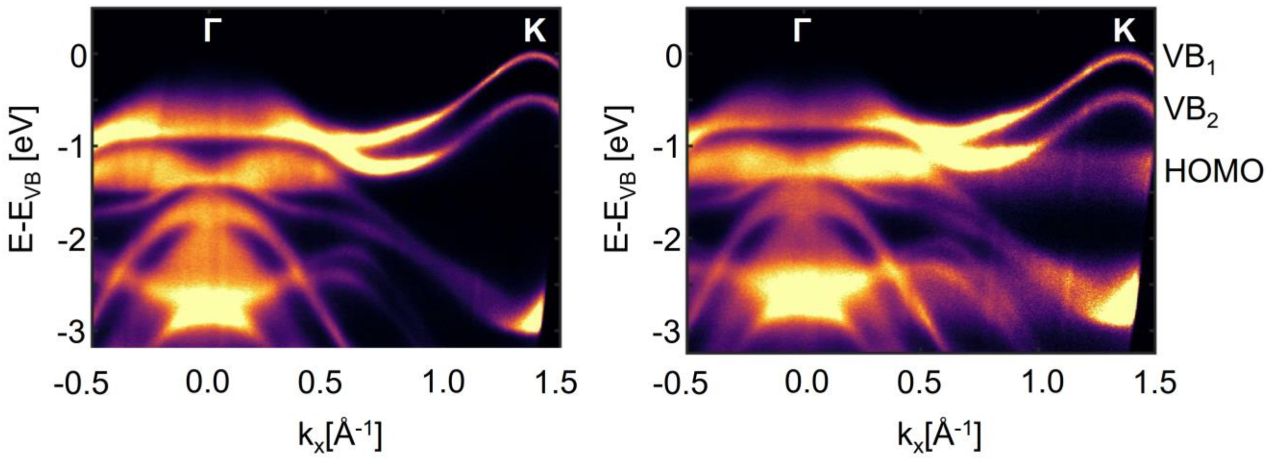

Fig. S1: Electronic valence band structure of WSe_2 and C_{60}/WSe_2.
Energy vs. momentum photoemission map of the bare WSe_2 crystal (left) and the C_{60}/WSe_2 heterostructure (right) along the Γ-K-direction (He I_α radiation). It shows the spin split WSe_2 valence bands with their hole-like dispersion (VB_1, VB_2) and the dispersion-less HOMO of C_{60}. No changes in the WSe_2 band structure can be observed upon the adsorption of C_{60}.

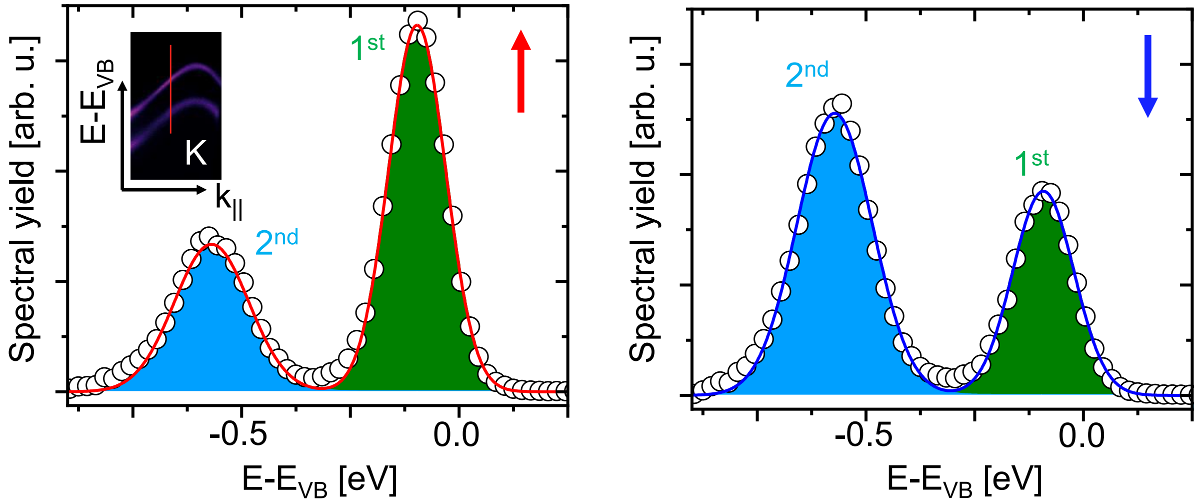

Fig. S2: Spin-polarized photoemission yield of the 2H-WSe_2 valence band structure. Spin-resolved photoemission yield (out-of-plane spin component) of the valence band structure obtained at a selected electron momentum (see red line in the inset of the left panel). The red and blue curves represent the fit to the spin-up and spin-down spectra, respectively. The contributions of the first and second layer valence bands to the spectral yield are fitted and plotted as green and blue Gaussian curves below the spectra. Our data analysis reveals a splitting of both valence bands of (470 ± 20) meV, which is in agreement with previous photoemission studies [1]. The different photoemission intensity of the valence band of the first and second WSe_2 layer is due to the small elastic mean free path of the photoelectrons of approx. 1 nm at small kinetic energies leading to an exponential attenuation of the photoemission signal with increasing distance from the surface [1,2].

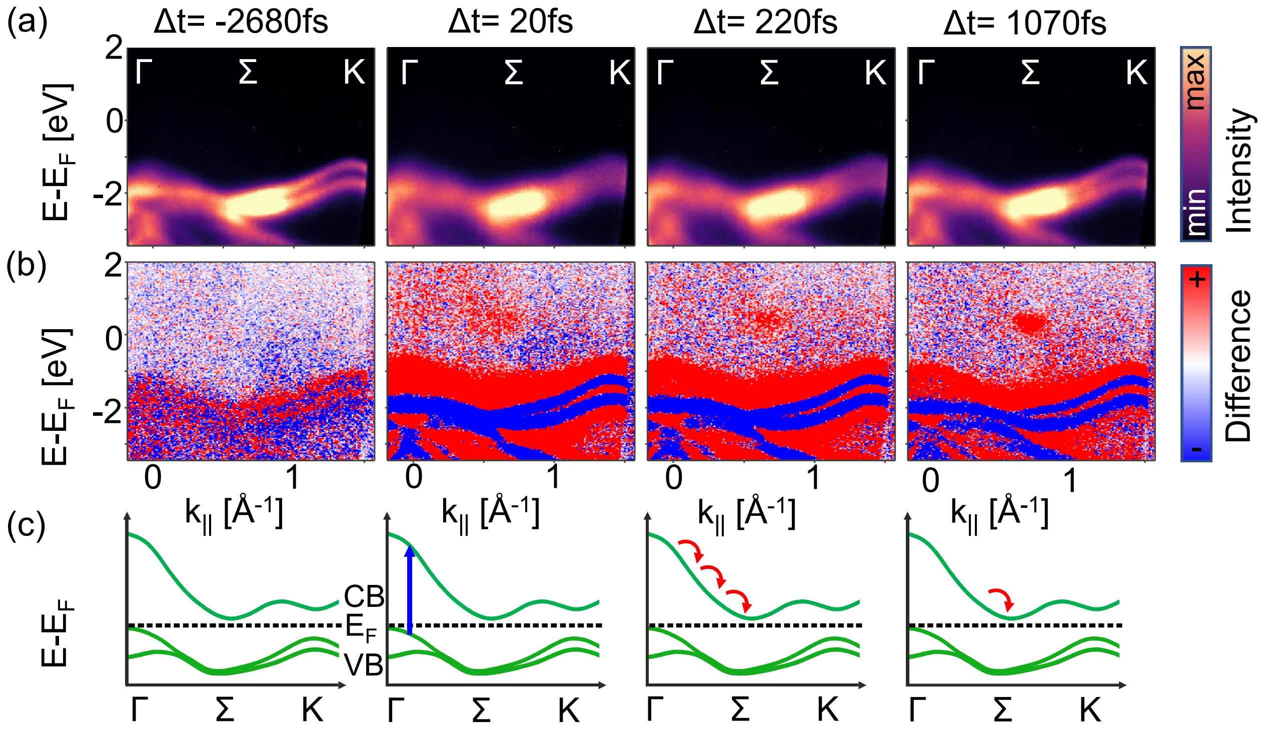

Fig. S3: Ultrafast dynamics of the bare WSe_2 centrosymmetric bulk crystal.
(a) Example energy vs. momentum intensity maps at selected time delays after optical excitation of a freshly cleaved WSe_2 bulk crystal with 3.2 eV photons (applied fluence $F = 0.5$ mJ/cm^2). This applied fluence is larger than that used to determine the single particle dynamics of hot carriers in TMD bulk crystals. However, it is still below the so-called critical Mott density, where excitons are suppressed [3]. Most importantly, the observed hot electron dynamics in the WSe_2 conduction band at this large fluence qualitatively mirrors the previously reported hot carrier dynamics of WSe_2 bulk crystals recorded for significantly smaller applied fluences [4]. The photoemission data around the Γ point were obtained with an angle of incidence of the pump and probe beam of 45°, the data at the K-point in normal incidence geometry. The WSe_2 valence bands show a substantial transient linewidth broadening, previously reported by M. Puppin et al. [3], which was attributed to an increased phase space for electron-electron scattering due to the depopulation of the WSe_2 valence bands. (b) Intensity difference maps of the same energy-momentum maps shown in (a). The difference maps were calculated by subtracting an intensity map before optical excitation (averaged over several time steps) from the intensity map at a selected time delay. In these difference maps, red areas indicate accumulation of spectral intensity, while blue areas indicate loss of spectral intensity. Changes in the excited states can be directly related to the population dynamics of the optically excited carriers. For the bare WSe_2 crystal, we observe an instantaneous population of the conduction band followed by intraband scattering of the carriers towards the Σ-valley, as shown in the energy level diagram in (c). In particular, we do not observe a strong population of the K-valley of the conduction band after optical excitation with 3.2 eV photons. This is significantly different from the situation of the

C_{60}/WSe_2 heterostructure discussed in the main manuscript as well as in Fig. S4. Our results are thus in qualitative agreement with the recent work of Puppin et al. for a bare 2H-WSe_2 bulk crystal [3,4].

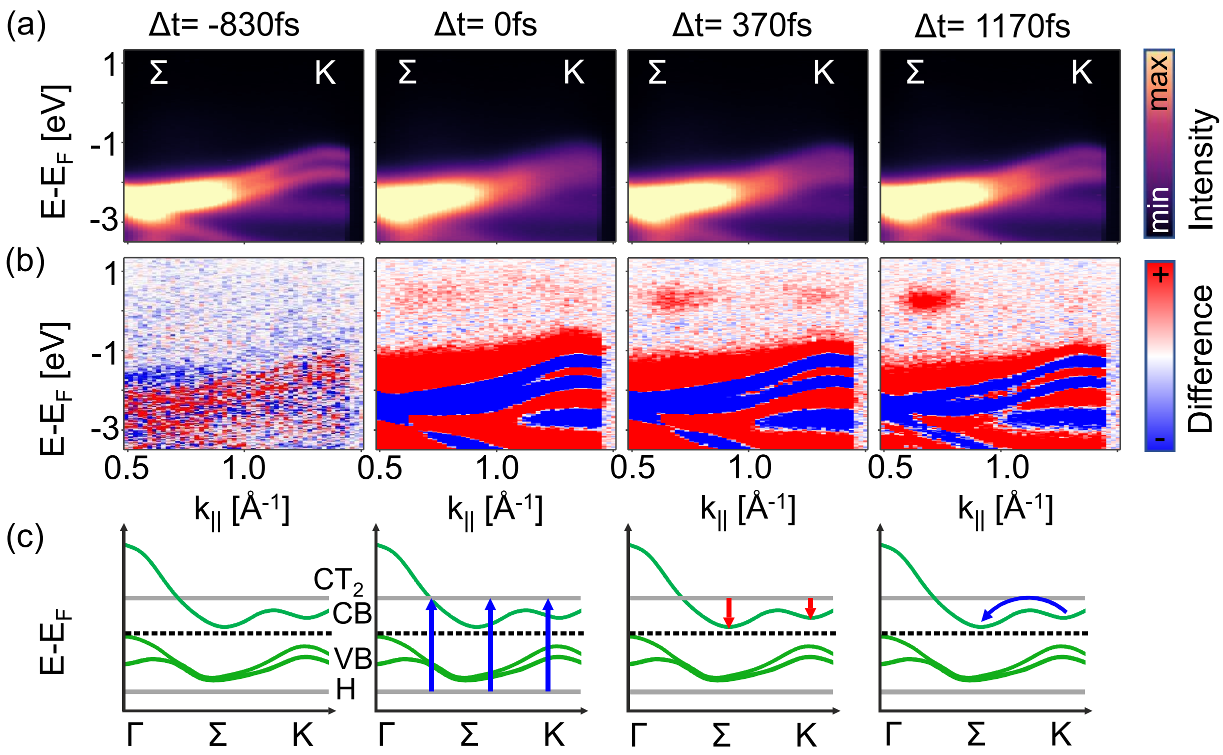

Fig. S4: Ultrafast dynamics of the C_{60}/WSe_2 heterostructure.
(a) Example energy vs. momentum intensity maps at selected time delays after optical excitation of an ultrathin C_{60} film grown on a freshly cleaved WSe_2 bulk crystal with 3.2 eV photons (applied fluence $F = 0.5$ mJ/cm^2). This rather large fluence is necessary to create the required charge density at the interface for the transient manipulation of the interfacial band structure. The photoemission data were obtained in the normal incidence geometry of the pump and probe beams (p-polarization). (b) Intensity difference maps of the energy-momentum maps shown in (a). The difference maps were calculated by subtracting an intensity map before optical excitation (averaged over several time steps) from the intensity map at a selected time delay. In these difference maps, red areas indicate accumulation of spectral intensity, while blue areas indicate loss of spectral intensity. The carrier dynamics are discussed in the main manuscript and illustrated in the energy level diagram in (c). In contrast to the photoemission data for the bare 2H-WSe_2 bulk crystal in Fig. S2, we find a clear population at the K-valley of the WSe_2 conduction band due to interlayer charge transfer from the optically excited C_{60} layer. This additional comparison between the raw data of bare and C_{60} covered WSe_2 provides further evidence for the ultrafast interlayer charge transfer from C_{60} into the first WSe_2 layer, as discussed in the main manuscript.

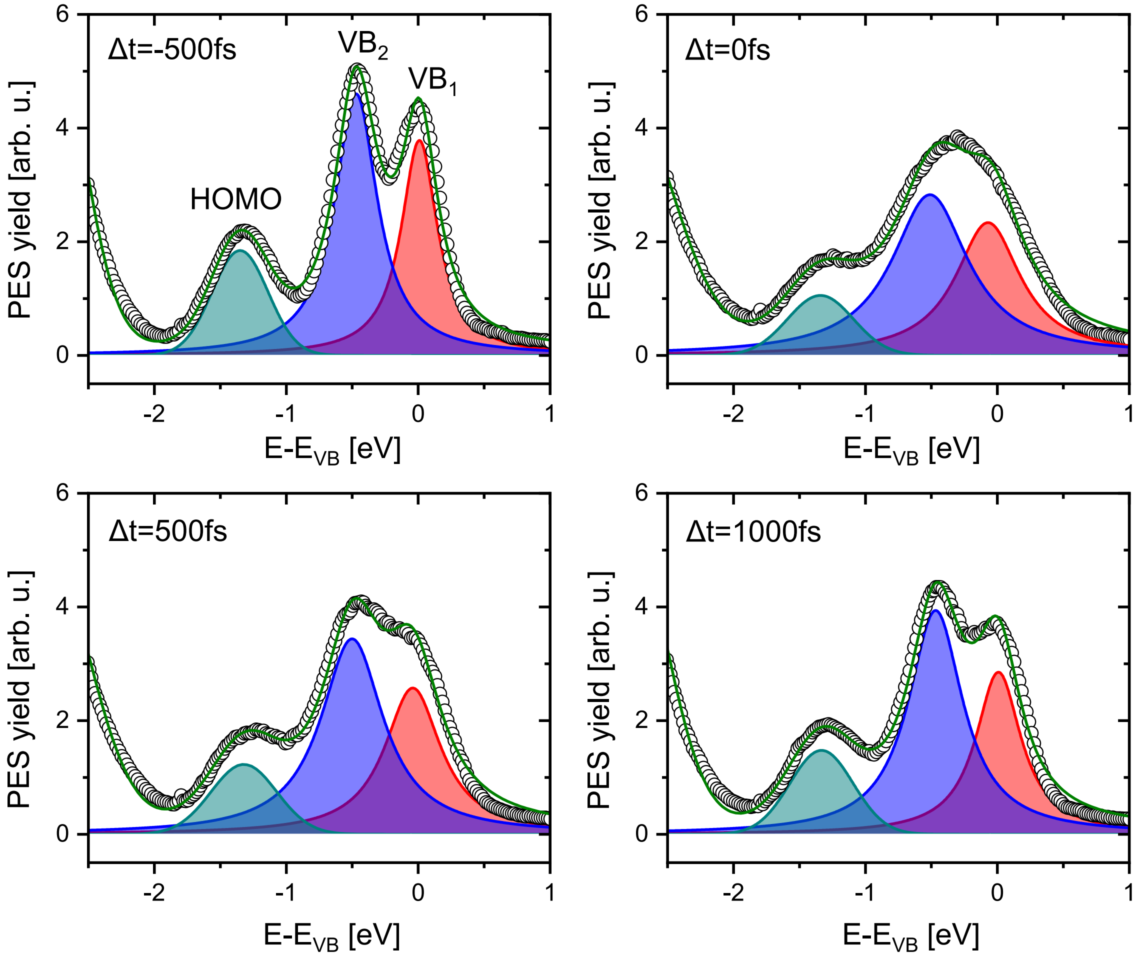

Fig. S5: Fitting model for the time-and momentum-dependent photoemission data of the C_{60}/WSe_2 valence band structure.

The data acquisition of the photoemission yield was performed in the snapshot mode of our photoemission analyzer system using a pass energy of 100 eV. The time-dependent changes of the photoemission signal were obtained by recording a 2D photoemission image (energy vs. emission angle) for each time delay between the 3.2 eV pump and the XUV probe pulse, see Fig. S4. The delay scans were repeated several times to improve the signal-to-noise ratio of the experimental data set. The 1D photoemission spectra of the occupied valence band region were extracted for each time delay from a small angular range (about 2°) around the K-point of the WSe_2 Brillouin zone. The analysis of these 1D spectra was performed separately for each spectrum of each time delay scan in two subsequent steps. First, the secondary electron background was subtracted from each spectrum using a Tougaard background [5]. To avoid artifacts in the subsequent fitting procedure, the background subtraction parameters were kept constant for each spectrum of a single delay scan. Example spectra after background subtraction are shown for four selected time delays. A spectral analysis was then performed to quantify the relative changes in peak area A, peak position $E\text{-}E_{VB}$ and linewidth (FWHM) for each spectroscopic feature, using a dedicated fitting model. The best fit was obtained by the model shown in this figure. The spectroscopic features of the spin-split WSe_2 valence bands (VB_1 and

VB_2) were modeled with Lorentzian functions, the molecular signals (HOMO and HOMO-1) with a Gaussian function.

The best fitting model is plotted as colored curves below the experimental data. Note that the signature of HOMO-1 is outside the energy range of the spectra shown in Fig. S5. The parameters of our fitting model were optimized by fitting the photoemission data at Δt = -500 fs. This optimized fitting model was propagated to all spectra of the delay scan using a minimum number of fitting constraints. The energy difference between the spin-split valence bands of WSe_2 (bands labeled VB_1 and VB_2) was fixed to (0.46 ± 0.03) eV, the maximum FWHM of the C_{60} HOMO was set to (0.16 ± 0.1) eV, and the FWHM of both WSe_2 valence bands was limited to be smaller than 0.3 eV. Using these constraints, we were unable to detect any signature of optically induced depletion of the WSe_2 valence band after excitation of the C_{60}/WSe_2 heterostructure with 3.2 eV pump pulses (see Fig. S6). Therefore, we additionally constrain the range of the Lorentzian functions modeling VB_1 and VB_2 to ± 10% of the area obtained for our fit at Δt = -500 fs. The fitting analysis yields the transient binding energy position and FWHM of the WSe_2 valence bands, as well as the binding energy position, FWHM, and area of the C_{60} HOMO. The fitting results of this data analysis procedure are shown in Fig. 2c, and Fig. 3c of the main manuscript and Figs. S5 and S6 of the supplemental material.

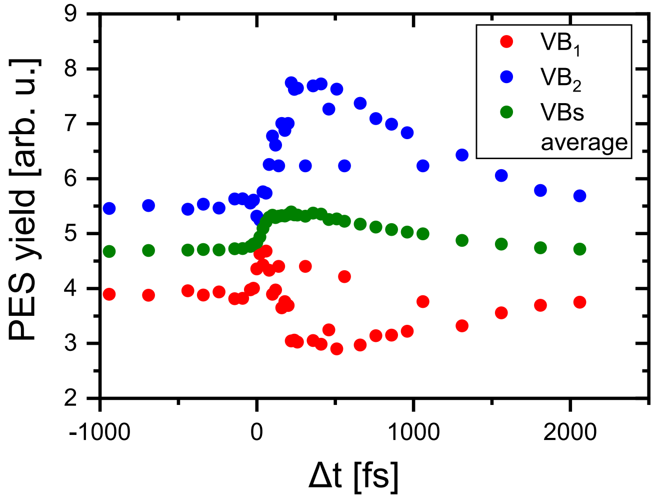

Fig. S6: Fitting result for the C_{60}/WSe_2 valence band structure – The area of the WSe_2 valence bands.

To quantify a potential depopulation of the WSe_2 valence band after optical excitation with 3.2 eV photons, we plot here the temporal evolution of the area of the Lorentzian functions modeling the valence bands of WSe_2. The results of our fitting model suggest a decrease in the intensity of VB_1 (red dots) within about 300 fs (i.e., on timescales significantly longer than the duration of the pump pulses) and a substantial increase in the intensity of VB_2 (blue dots). In total, this leads to an apparent overall small increase in the intensity of the WSe_2 valence bands (green dots). The opposite intensity changes of VB_1 and VB_2 do not indicate a substantial depopulation of VB_1, but rather an imperfect separation of the time-dependent spectral yields of VB_1 and VB_2. This is not surprising considering that the intensity changes coincide with the time scale of the transient linewidth broadening of the WSe_2 valence bands. As shown in Fig. S4, the transient linewidth broadening leads to a merging of the characteristic double peak structure of the spin-split WSe_2 valence bands into a single, nearly symmetric spectroscopic feature. Combined with the significantly different excited state dynamics of the bare WSe_2 crystal and the C_{60}/WSe_2 heterostructure, our line-shape analysis of the C_{60}/WSe_2 valence band structure points to at best small extent of optical excitation of carriers in the bulk WSe_2 crystal below the C_{60} layer.

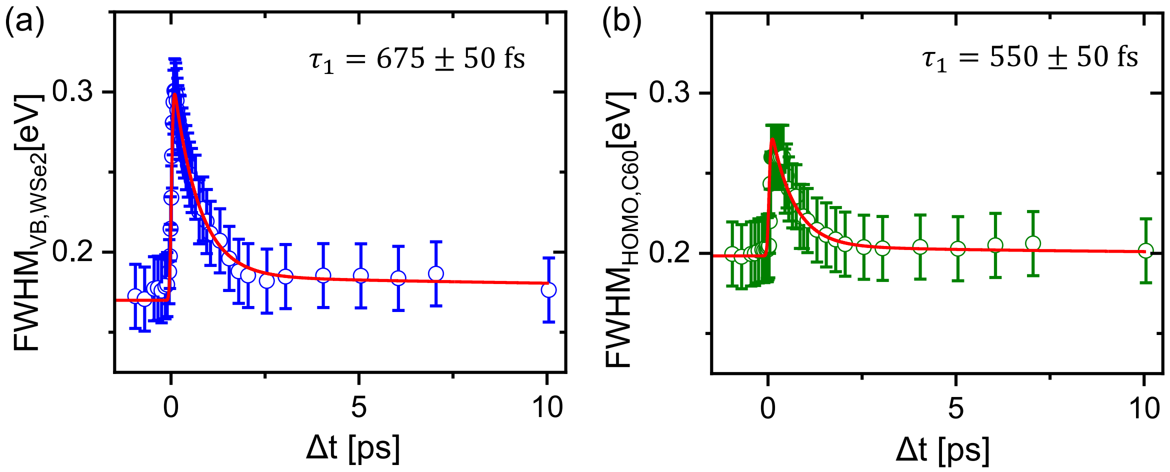

Fig. S7: Fitting result for the C_{60}/WSe_2 valence band structure – The transient linewidth broadening of the C_{60}/WSe_2 valence bands.

The temporal evolution of the transient linewidth broadening of the WSe_2 valence bands (a) and the C_{60} HOMO (b) were determined by the fitting model discussed in the caption of Fig. S5. The error bars estimate the uncertainty of the fitting procedure to the experimental data. The temporal evolution of these data was analyzed using a simple exponential fit function with a single decay time τ_1. This exponential function was convolved with a normalized Gaussian with a FWHM of Δt = 70 fs, and the resulting analytical function was fitted to the experimental data using a least-squares fitting procedure. We find a different temporal evolution of the transient linewidth broadening for the valence states in WSe_2 and the C_{60} layer within our experiment uncertainty.

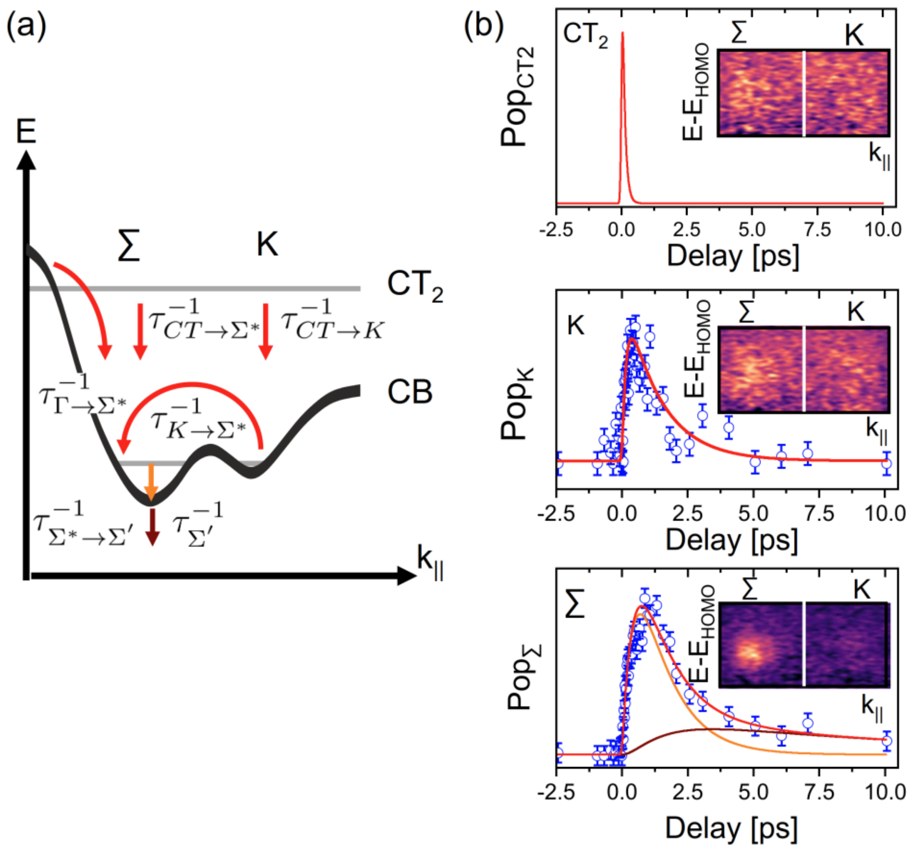

Fig. S8: Illustration of the rate equation model analysis of the excited state populations. (a) Excited state energy level alignment of the C_{60}/WSe_2 heterostructure. The scattering paths of our rate equation model are indicated by straight and curved arrows together with the corresponding scattering rates considered in the set of rate equations. (b) Comparison of the extracted photoemission yield in the excited states with the simulated time-dependent population traces of our rate equation model. The insets show example energy vs. momentum maps for a selected time during the population of the respective molecular or WSe_2 excited state. The vertical white line separates the momentum space region assigned to the excited state population of the Σ- (left side) and K-valley (right right). The excited population at selected time delay is determined by first numerically integrating the photoemission intensity in the corresponding momentum space region marked in the inset. The resulting excited state spectrum $I_{K/\Sigma}(E,t)$ is then background-corrected with a linear background, and finally fit by a single Gaussian function for each time delay. The intensity of this Gaussian function $A_{K/\Sigma}(t)$ reflects the transient excited state population of the Σ- and K-valley. Note that despite this dedicated fitting procedure, no clear population signal could be extracted for the CT_2 feature due to its broad emission pattern in momentum space as well as its energetic overlap with the WSe_2 conduction band.

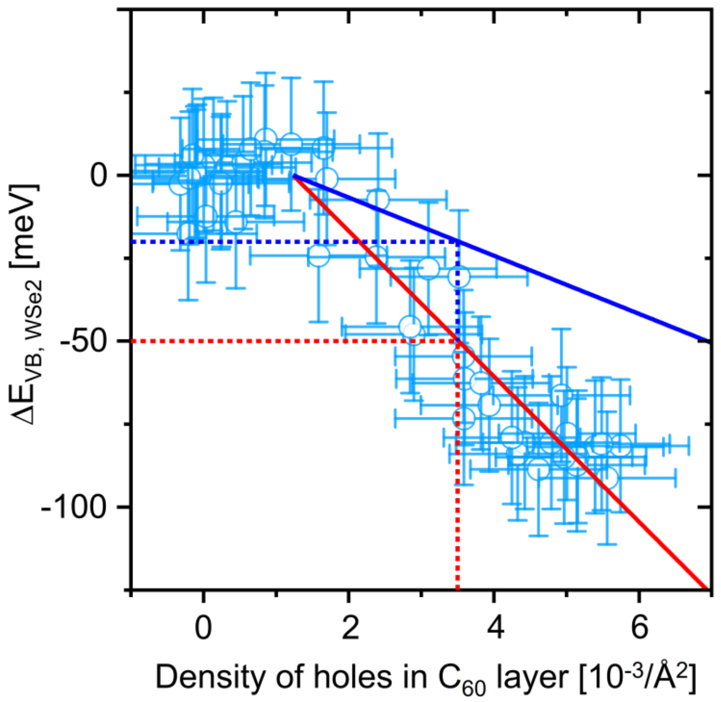

Fig. S9: Relationship between the optical charge doping of the C_{60} layer and the magnitude of the transient shift of the WSe_2 valence band structure.

The light blue data points show the correlation between the magnitude of the valence band shift of the first WSe_2 layer and the number of carriers in the C_{60} layer. The magnitude of the valence band shift was determined from the spectral analysis of the spin-integrated photoemission data presented in Fig. 3c of the main manuscript. These data mainly reflect the transient electronic properties of the first WSe_2 layer due to the small elastic mean free path of photoelectrons at low kinetic energies. The density of carriers (holes) in the C_{60} layer was determined from the transient loss of intensity of the HOMO signal shown in Fig. 2c of the main manuscript, the coverage of C_{60} of 0.8 ML, a maximum packing density of C_{60} molecules on surfaces of one C_{60} molecule per 100 Å^2, and an excitation density of 70% of all C_{60} molecules. The error bars were estimated and propagated from the results of the data analysis procedures presented in Figs. 2c and 3c of the main manuscript. We find a linear relationship between the hole density and the valence band energy shift of the first layer (red line), with a turn-off below 0.001 charges/Å^2. This linear relationship between charge density and valence band shift of the first WSe_2 layer, together with our spin- and time-resolved photoemission data (see Fig. 4 of the main manuscript), allows us to estimate the magnitude of the relative valence band shift of the first and second WSe_2 layers. For a given hole density in the C_{60} layer, we find a relative shift of the valence band of (50 ± 20) meV for the first (red dotted lines) and (20 ± 20) meV for the second WSe_2 layer (blue dotted lines) in our spin- and time-resolved photoemission experiment. Assuming a similar linear relationship between hole density and valence band shift for the second WSe_2 layer, as well as a similar turn-off hole density, we can estimate the transient valence band shift of the second WSe_2 layer, see blue solid line in Fig. S9. Accordingly, we find a linear increase in the spin splitting of the first and second layer WSe_2 valence band with

increasing hole density in the C_{60} layer, as quantified by
$\Delta E_{\mathrm{VBs}} = -13.15\ \text{Å}^2 \cdot \mathrm{eV} \cdot \rho_{\mathrm{holes}} + 0.0162\ \mathrm{eV}$

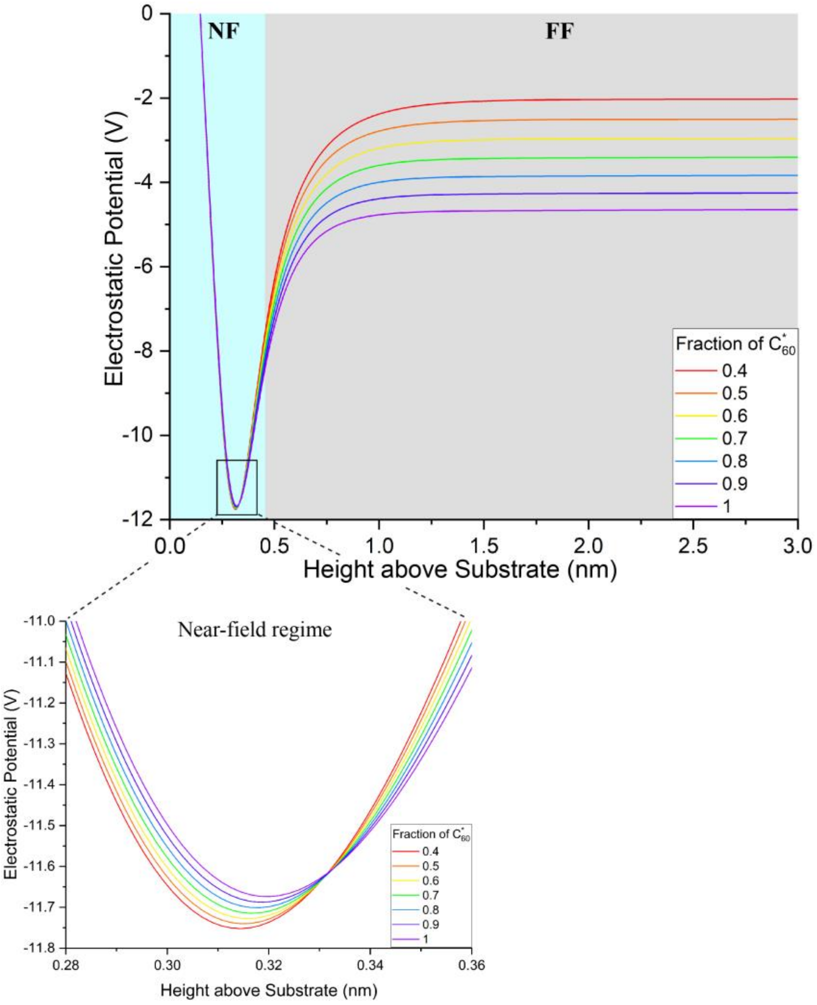

Fig. S10: Electrostatic potential as a function of height above substrate.
The blue shaded region is the near-field region (NF), and the grey-shaded region is far-field (FF). The inset shows the switch from NF to FF behavior, where increased excitation densities increase the electrostatic potential within the physical dipole.

Supplementary Methods

A. Rate Equation Model:

The temporal evolution of the charge carriers was modeled by solving a rate equation model for the excited state populations P_i of the molecular CT_2 state (i = CT), the population at the K-valley of WSe_2 (i = K), the population of the WSe_2 conduction band between the Γ-point and the Σ-valley (i = Γ), the high energy population at the Σ-valley ($i = \Sigma^*$) as well as at the band bottom of the Σ-valley (i = Σ'). The population of the WSe_2 conduction band between the Γ-point and the Σ-valley was considered to account for the intrinsic charge carrier dynamics of WSe_2 after optical excitation with 3.2 eV photons. In our simulations, the initial population is created in the molecular state CT_2 as well as in the WSe_2 conduction band between the Γ-point and the Σ-valley using the laser light field $L(t)$ that is partially absorbed in the molecular and the WSe_2 layer. The laser light field is modeled as a delta pulse at t = 0 fs and the parameter a determines the relative efficiency of the light absorption in both layers. The carrier scattering is assumed to be mono-exponential with rates that are given by the inverse of the scattering times τ_i. All possible scattering pathways are illustrated in Fig. S8(a) and the set of rate equations are:

$$\frac{\mathrm{d}P_{\mathrm{CT}}}{\mathrm{d}t} = -\frac{1}{\tau_{\mathrm{CT}\to\mathrm{K}}} \cdot P_{\mathrm{CT}}(t) - \frac{1}{\tau_{\mathrm{CT}\to\Sigma*}} \cdot P_{\mathrm{CT}}(t) + a \cdot L(t)$$

$$\frac{\mathrm{d}P_{\mathrm{K}}}{\mathrm{d}t} = \frac{1}{\tau_{\mathrm{CT}\to\mathrm{K}}} \cdot P_{\mathrm{CT}}(t) - \frac{1}{\tau_{\mathrm{K}\to\Sigma*}} \cdot P_{\mathrm{K}}(t)$$

$$\frac{\mathrm{d}P_{\Sigma*}}{\mathrm{d}t} = \frac{1}{\tau_{\mathrm{CT}\to\Sigma*}} \cdot P_{\mathrm{CT}}(t) + \frac{1}{\tau_{\mathrm{K}\to\Sigma*}} \cdot P_{\mathrm{K}}(t) + \frac{1}{\tau_{\Gamma\to\Sigma*}} \cdot P_{\Gamma}(t) - \frac{1}{\tau_{\Sigma*\to\Sigma'}} \cdot P_{\Sigma*}(t)$$

$$\frac{\mathrm{d}P_{\Sigma'}}{\mathrm{d}t} = \frac{1}{\tau_{\Sigma*\to\Sigma'}} \cdot P_{\Sigma*}(t) - \frac{1}{\tau_{\Sigma'}} \cdot P_{\Sigma'}(t)$$

$$\frac{\mathrm{d}P_{\Gamma}}{\mathrm{d}t} = -\frac{1}{\tau_{\Gamma\to\Sigma*}} \cdot P_{\Gamma}(t) + (1-a) \cdot L(t)$$

The sum of $P_{\Sigma'}(t)$ and $P_{\Sigma*}(t)$ corresponds to the total population of electrons at the Σ-valley of WSe_2. However, this separation of the population at the Σ-valley into two contributions reflects

the intravalley carrier scattering reported by Puppin et al. [3,4] and improves the quality of our data analysis procedure.

To quantify the scattering time, we first analytically solved this set of coupled differential equations and convoluted the resulting time traces with a normalized Gaussian function (FWHM of Δt = 70 fs) to account for the temporal broadening of our experimental data. Afterwards, we manually fitted the analytical functions for the population of $P_{CT}(t)$, $P_K(T)$, and $P_\Sigma(t) = P_{\Sigma'}(t) + P_{\Sigma*}(t)$ to the experimental data. For comparison with the measured photoemission intensities Pop_i, we used a weighting factor α_i: $Pop_i(t) = \alpha_i P_i(t)$. The results are shown in Fig. 2c of the main manuscript and in the supplementary figure S8(b). The most consistent fitting result was obtained for $\tau_{CT\rightarrow\Sigma*} = \tau_{CT\rightarrow K}$, i.e., we assume an identical charge transfer time from the molecular layer into both valleys of the WSe_2 conduction band. In addition, we find a substantially more efficient charge transfer from the CT_2 state into the K- than into the Σ-valley (90% vs. 10%). Finally, we would like to emphasize that the scattering time $\tau_{\Gamma\rightarrow\Sigma*}$ could only be determined indirectly by the shape of the population dynamics at the Σ-valley. The best fitting result was obtained for $\tau_{\Gamma\rightarrow\Sigma*} = 1.1ps$.

B. Electrostatic Model Simulations

We used a simple electrostatic model to determine the magnitude and direction of the transient electric fields due to charge transfer at the interface of C_{60} and WSe_2. Beyond a minor static interfacial dipole, an additional electrostatic potential step emerges as a result of interfacial charge transfer from C_{60} to WSe_2. We model the magnitude of this transient electrostatic potential by an array of physical dipole moments μ per area, A, by the Helmholtz equation:

$$\Delta E = \frac{\mu}{\epsilon_0 A} = \frac{q_\mathrm{e} d}{\epsilon_0 A}$$

where $\epsilon_0$is the vacuum permittivity, and q_e the charge separated by distance, d. By considering a finite array of dipole moments made up of point charges, we model the electrostatic potential of this interface.

From STM data of C_{60} on WSe_2, the reported height of C_{60} on WSe_2 is approximately 1 nm, and the radius of C_{60} is 7.1 Å. [6] We assume that the holes, or array of positive charges, are localized at the bottom of the C_{60} cage, while the electrons, or negative charges, are confined to the top-most layer of WSe_2, specifically the top-most Se-atom. Due to the van der Waals nature of interlayer binding in WSe_2, we do not expect lower layers in WSe_2 to participate significantly in the formation of the electrostatic field. The resulting charge separation distance of 2.9 Å establishes electrostatic potentials that qualitatively reproduce the observed maximum transient energy shifts. In what follows, we discuss all aspects of our model and built-in assumptions.

We estimate the excitation densities from the transient depopulation of the HOMO level of C_{60} at key time-steps. Optical excitation of C_{60} with 3.2 eV sub-50 fs pulses resonantly excites electrons from the HOMO level into the LUMO+1* level, leading to the formation of a charge transfer exciton in the C_{60} layer, the CT_2 state. [7] The HOMO feature shows an instantaneous intensity reduction of approximately 35%. At the high fluences used in our experiments and based on the photoemission cross section of the C_{60} HOMO bands for s-polarized light [8], we estimate an excitation efficiency of about 80% of all C_{60} in the thin film.

Based on the high density of electron-hole pairs upon interfacial charge transfer, additional effects need to be taken into account for estimating the resulting potentials. Blumenfeld et al. showed previously for thin films of molecules supporting a permanent dipole moment that at high enough dipole densities depolarization effects strongly impact the interfacial electrostatics [9]. Depolarization results from the fact that each dipole moment in the array induces a dipole moment in the surrounding molecules that is aligned in the opposite direction, thus reducing the transient dipole moment. Following their model, the effective transient dipole moment, $\tilde{\mu}_{ind}$ including depolarization effects can thus be expressed as:

$$\tilde{\mu}_{\text{ind}} = \frac{\mu_{\text{ind}}}{1 + f\tilde{\alpha}_{zz}\rho_{\text{dip}}^{3/2}}$$

where $\rho_{\text{dip}}$is the density of transient dipoles, f is a geometric factor capturing the geometric arrangement of dipole moments on the surface and known as the Topping constant [10], and $\tilde{\alpha}_{zz}$ is the *zz*-component of the effective polarizability tensor. We estimate the latter based on the known static polarizability of C_{60} $\alpha_{zz} = 8 \cdot 10^{-29}$ m^3 [11], and consider screening effects by both sides of the interface by including their respective dielectric components into the simulation. The static dielectric constant of C_{60} is $\epsilon_{\text{C60}} = 4.5$ and the bulk out-of-plane component of the static dielectric constant for WSe_2 is $\epsilon_{\text{WSe2}} = 7.8$ [11,12]. This yields the *zz*-component of the effective polarizability tensor $\tilde{\alpha}_{zz}$:

$$\tilde{\alpha}_{zz} = \frac{\alpha_{zz}}{4\pi\epsilon_0\epsilon_{\text{avg}}}$$

where ϵ_{avg} is the average dielectric constant of the two materials. By way of comparison, we estimate an induced dipole moment of approximately 13.9 D for a charge separation of 2.9 Å.

The electrostatic potential as a function of height above the surface is shown in Figure S10 for different fractions of excited C_{60} molecules. From the electrostatic simulations, we identify two regimes of the electrostatic surface potential (near- and far-field, highlighted in blue and grey, respectively). At approximately 3.3 Å, we observe an isosbestic-like point where the far-field effects begin to dominate. In the far-field regime, the electrostatic potential above the dipole array decays rapidly, converging to a constant potential step, representing a transient change of the global work function of the system as a result of charge-separation. As expected and given the sign of the dipole moment, an increase in excitation density (charge density) on the surface decreases the electrostatic potential and therefore the work function.

The near-field potential, or the potential "inside" the physical dipole, gives rise to an electrostatic potential whose behavior contrasts with the far-field potential: Due to depolarization, the electrostatic potential decreases with increasing charge densities. The electrostatic potential in the near-field regime explains therefore both signs and magnitude of the Stark shifts observed in the C_{60} HOMO and WSe_2 valence band: At lower charge densities, the magnitude of the electrostatic potential close to the dipole array increases.

Supplementary References

[1] J. M. Riley et al., *Direct Observation of Spin-Polarized Bulk Bands in an Inversion-Symmetric Semiconductor*, Nat. Phys. **10**, 835 (2014).

[2] B. Parashar et al., *Photoemission study of twisted monolayers and bilayers of* WSe_2 *on graphite substrates*, Phys. Rev. Mater. **7**, 044004 (2023)

[3] M. Puppin, *Time- and Angle-Resolved Photoemission Spectroscopy on Bidimensional Semiconductors with a 500 KHz Extreme Ultraviolet Light Source*, Free University of Berlin, 2018.

[4] M. Puppin et al., *Excited-State Band Structure Mapping*, Phys. Rev. B **105**, 075417 (2022).

[5] S. Tougaard, *Practical Guide to the Use of Backgrounds in Quantitative XPS*, J. Vac. Sci. Technol. A **39**, 011201 (2021).

[6] E. J. G. Santos, D. Scullion, X. S. Chu, D. O. Li, N. P. Guisinger, and Q. H. Wang, *Rotational Superstructure in van Der Waals Heterostructure of Self-Assembled* C_{60} *Monolayer on the* WSe_2 *Surface*, Nanoscale **9**, 13245 (2017).

[7] B. Stadtmüller et al., *Strong Modification of the Transport Level Alignment in Organic Materials after Optical Excitation*, Nat. Commun. **10**, 1470 (2019).

[8] N. Haag et al., *Signatures of an Atomic Crystal in the Band Structure of a* C_{60} *Thin Film*, Phys. Rev. B **101**, 165422 (2020).

[9] M. L. Blumenfeld, M. P. Steele, and O. L. A. Monti, *Near- and Far-Field Effects on Molecular Energy Level Alignment at an Organic/Electrode Interface*, J. Phys. Chem. Lett. **1**, 145 (2009).

[10] J. Topping, *On the Mutual Potential Energy of a Plane Network of Doublets*, Proc. R. Soc. London. Ser. A, Contain. Pap. a Math. Phys. Character **114**, 67 (1927).

[11] R. R. Zope, T. Baruah, M. R. Pederson, and B. I. Dunlap, *Static Dielectric Response of Icosahedral Fullerenes from* C_{60} *to* C_{2160} *Characterized by an All-Electron Density Functional Theory*, Phys. Rev. B **77**, 115452 (2008).

[12] A. Laturia, M. L. Van de Put, and W. G. Vandenberghe, *Dielectric Properties of Hexagonal Boron Nitride and Transition Metal Dichalcogenides: From Monolayer to Bulk*, Npj 2D Mater. Appl. **2**, 6 (2018).
